# Clinical and radiological evaluation of surgical treatment of medial epicondyle fractures of humerus in children. A two-centre study

**DOI:** 10.1038/s41598-023-37063-7

**Published:** 2023-06-21

**Authors:** Wojciech Stelmach, Jacek Beczkowski, Piotr Zając, Krzysztof Małecki, Marcin Sibiński, Kryspin Niedzielski

**Affiliations:** 1 Clinic of Orthopaedic and Traumatology, Polish Mother’s Memorial Hospital Research Institute, Lodz, Poland; 2 Department of Orthopaedics, Świetokrzyskie Pediatric Center, Regional Hospital, Kielce, Poland; 3 Clinic of Orthopaedics and Paediatric Orthopaedics, Medical University of Lodz, 92-213 ul. Pomorska 251, Lodz, Poland

**Keywords:** Health care, Medical research

## Abstract

The present study analyses the outcome of open reduction and internal fixation (ORIF) of humerus medial epicondyle fracture with the use of Kirschner (K) wires, and determine the effect of elbow dislocation. The study included 112 patients operated on in 2005–2016. Of these, 81presented with an isolated medial epicondyle fracture (mean age 11.6 years), and 31 with an elbow dislocation (mean age 11.9 years). Out of 112 patients tested, 98 achieved an excellent treatment result, ten good and a mean Mayo Elbow Performance Score (MEPS); no significant differences were observed between dislocated and non-dislocated elbow groups. Those with an isolated medial epicondyle fracture demonstrated a mean flexion of 140.7° and extension deficit of 3.0°, while those with an elbow dislocation displayed a mean flexion of 134.5° and extension deficit 6.1°. The dislocation group demonstrated significantly greater extension and flexion deficits (p = 0.019, p < 0.001, respectively). One patient required revision surgery due to nonunion. Ulnar nerve function was normal in 110 patients: in the other two, it resolved spontaneously in one, and the nerve was transposed in the other. Medial elbow instability was found in seven patients: two with elbow dislocation and five without. ORIF with K wires is a safe procedure for treating medial epicondyle humeral fractures that yields good or very good results. Similar outcomes are observed between patients with and without dislocation according to MEPS; however, flexion and extension are more limited in the former group.

## Introduction

In the paediatric population, medial epicondyle fractures account for 11–20% of all humerus fractures, with most occurring between the ages of 11 and 12^1–4^, and being four times as common among boys than girls. In 30–60% of cases, fracture is associated with elbow dislocation^5–11^. The medial epicondyle is the site of the attachment of various muscles, including the radial and ulnar flexor of the wrist, the superficial flexor of the fingers, the palmaris longus and the pronator teres muscle.

Although injury can occur directly, leading to multi-fragment fractures, but it is more commonly indirect; such cases often result in elbow deformation, leading to an avulsion fracture of the medial epicondyle or a sudden hyperextension causing dislocation of the elbow^12^. Diagnosis is based primarily on physical examination combined with radiological examination in the AP and lateral projection. X-ray images can be used to determine the degree and direction of the fracture displacement (according to the Watson-Jones classification), as well as the degree of displacement and the nature of the fracture (according to the Wilkins classification)^7, 13^.

Treatment of medial epicondyle fracture can be operative or conservative. Indications for surgical fixation for paediatric medial epicondyle fractures include: open fracture, concurrent elbow dislocation, fragment incarceration, fracture displacement > 5 mm, and fractures in upper extremity athletes^4, 6, 9, 14^. In surgical treatment, access is achieved from a medial incision with an ulnar port visualization. The fragments are typically joined using sutures, Kirschner wires or pulling screws, as well as bioabsorbable screws or anchors. However, the decision to choose surgical or conservative treatment, as well as the method of fixation, remains controversial^4, 6, 9, 14^. K- wires stabilization is the procedure performed in our centres. This is a widely available and cheap surgical approach to fixed bone fractures that does not require high technical skills.

The aim of the study was to analyse the outcomes of ORIF treatment for medial epicondyle fractures using Kirschner wires, and to compare the results between patients with and without elbow dislocation.

## Materials and methods

The study included 112 patients (39 girls and 73 boys; age 3 to 17 years) treated in two paediatric orthopaedics centres. All presented with a fracture of the medial epicondyle of the humerus, and were treated by surgery with Kirschner wire stabilization. All patients treated conservatively, and those with concomitant fractures of the humerus and forearm, were excluded from the study. The data are given in (Tables 1 and 2).

**Table 1:** Demographic information of the study subjects.

Side of the injured elbow (n)		Isolated medial epicondyle fracture (n)		Elbow dislocation and medial epicondyle fracture (n)		Ulnar nerve disfunction (n)
Right 62	Left 50	Right side 47	Left side 34	Right side 15	Left side 16	2 patients
		28 girls	53 boys	11 girls	20 boys

**Table 2:** Patients age and follow- up information.

Isolated medial epicondyle fracture	Elbow dislocation and medial epicondyle fracture
Mean age 11.6 years (range 3 to 17 years)	Mean age 11.9 years (range 5 to 16 years)
The mean follow-up time 26.3 months (range 8 to 87 months)	The mean follow-up time 29.9 months (range 12 to 72 months)

All patients were treated surgically. The surgical procedure was performed with tourniquet from medial surgical approach (ORIF). The fractures were internally fixed after dissecting of the tissue and identification of the ulnar nerve with 40°–60° flexion in the elbow joint with concomitant pronation of the forearm. We used two or three Kirschner wires followed by plaster cast immobilization. In patients with elbow dislocation, a closed reduction was first performed, followed by an open epicondyle repositioning with Kirschner wire stabilization. The fragments of the bones were assessed intraoperatively by fluoroscopy. The joint was immobilized for three weeks in the case of a fracture with dislocation, and five weeks in the case of an isolated fracture. In each case, the number of wires used was dictated by the size of the detached bone fragment, which ranged from 11 × 6 to 20 × 10 mm, measured intraoperatively. After the plaster immobilization was removed, the elbow joint improved until a satisfactory range of motion was obtained.

The range of motion was measured with a goniometer. The valgus of the elbow was assessed using the RadiAnt DICOM Viewer programme based on X-ray images (AP projection). Each patient was assessed for elbow stability and UCL (ulnar collateral ligament) performance using the valgus stress test.

In all patients, the degree of fracture of the medial epicondyle was assessed based on X-ray images taken before surgery according to the Watson-Jones^13^ and Wilkins classifications^7^ using computer programme RadiAnt DICOM Viewer. According to Watson- Jones Classification we measured the distance of bone fragment transposition in the AP projection of the elbow^13^. Based on the displacement of the fracture we classified the fracture into the appropriate group.

Watson–Jones classification^13^

- Type 1- Fracture with displacement less than 5 mm without rotation.
- Type 2- Fracture with displacement less than 5 mm with rotation.
- Type 3- Fracture with a trapped fragment without dislocation.
- Type 4- Fracture with a trapped fragment with dislocation.

Wilkins classification^7^

- Fracture not displaced
- Fracture slightly displaced
- Significantly displaced fracture (with or without an elbow dislocation)
- Fragmented fragment in the elbow joint (with or without an elbow dislocation)
- Fracture by apophysis of the medial epicondyle (with or without displacement)

Treatment outcome was assessed according to a point scale of elbow joint functionality, i.e. Mayo Elbow Performance Score (MEPS). The data are given in Table 3.

**Table 3:** Elbow functionality scale—Mayo elbow performance score. Patient outcomes.

Pain	None	99 patients
	Mild	13 patients
	Moderate	0 patients
	Severe	0 patients
Motion	> 100°	107 patients
	50–100°	5 patients
	< 50°	0 patients
Stability	Stable	105 patients
	Moderate instability	7 patients
	Gross instability	0 patients
Function	25 scores	99patients
	20 scores	9 patients
	15 scores	4 patients
	10 scores	0 patients
	5 scores	0 patients

**Figure 1:**
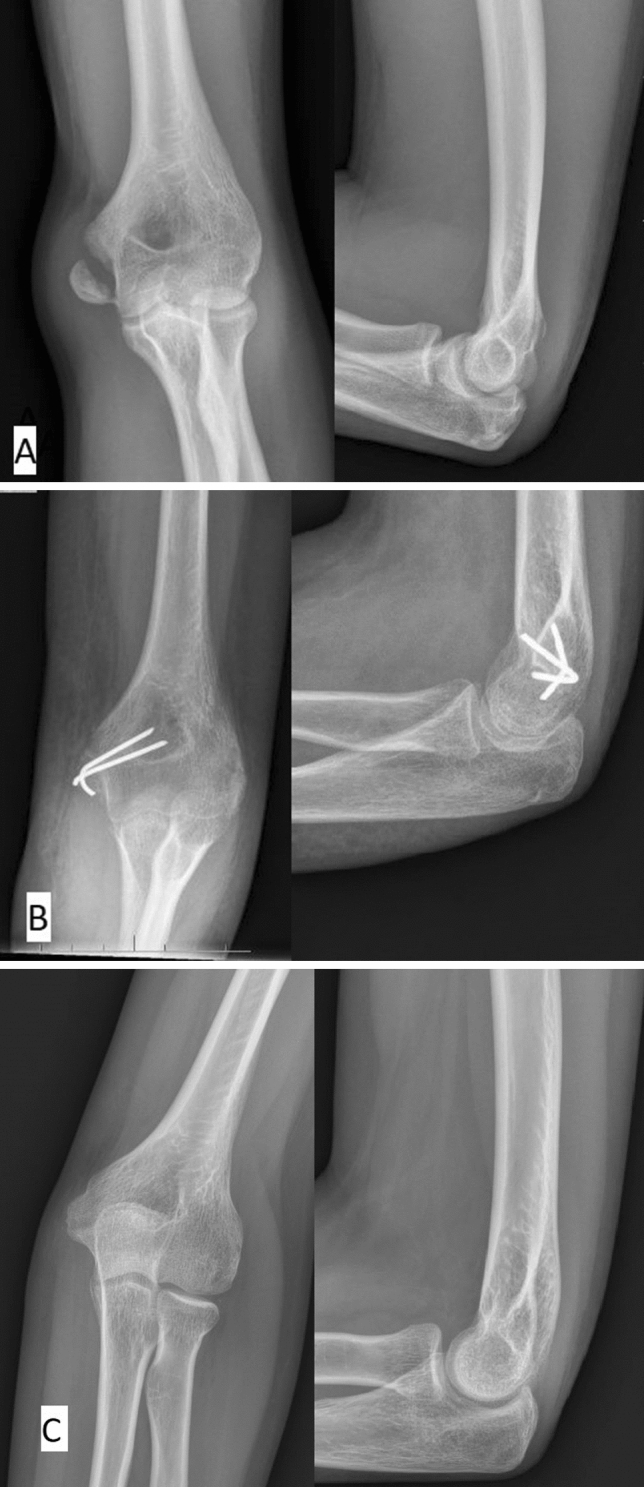
(**A**) Thirteen-year-old girl with isolated medial epicondyle fracture. (**B**) Six weeks after surgery. (**C**) Four years after surgery.

**Figure 2:**
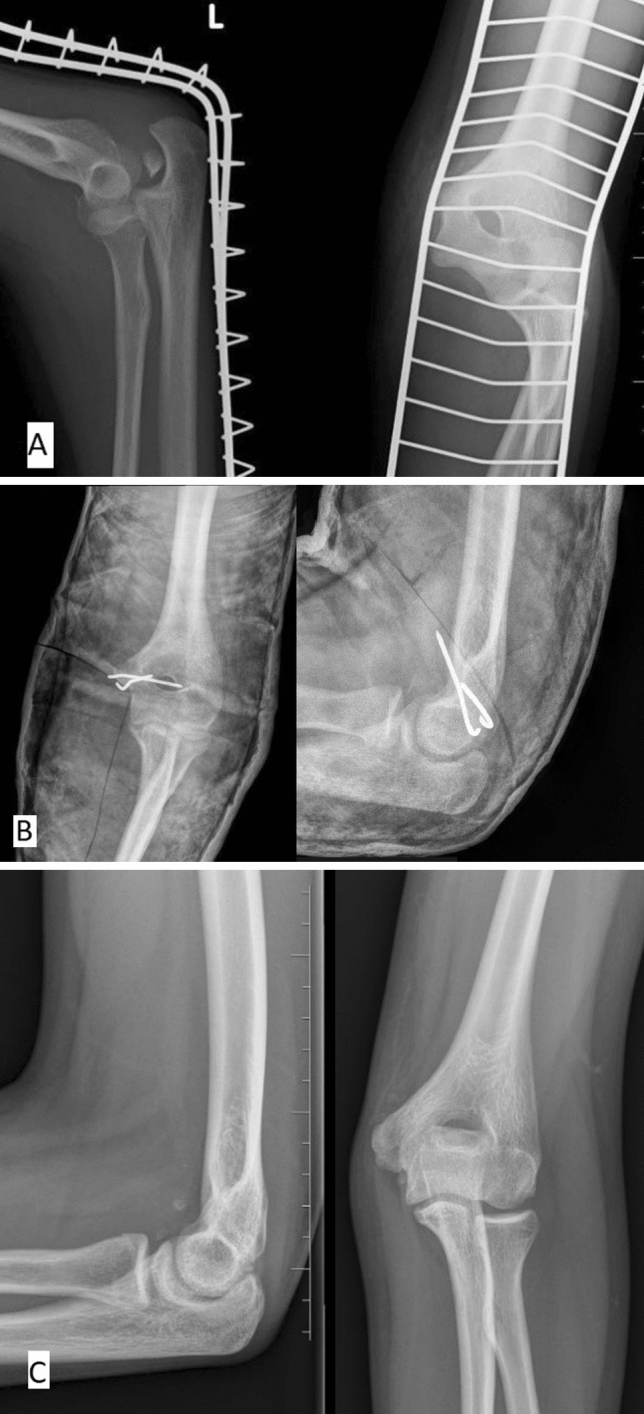
(**A**) Thirteen-year-old boy demonstrating medial epicondyle fracture with associated intra- articular displacement and elbow dislocation. (**B**) Patient 6 weeks after surgery. (**C**) Patient 2 years after surgery. Perfect outcome.

The fracture and radiographic results of the treatment with isolated displacement is shown in Fig. 1A–C, while the fracture and results of the treatment with elbow dislocation is shown in Fig 2A–C.

The study was approved by Bioethical Committee at Polish Mother's Memorial Hospital Research Institute (project number 8/2020). This study was performed in line with the principles of the Declaration of Helsinki. Informed consent all have been obtained from a parent and/or legal guardian of participants included in the study. If patients were 13 years old or more the informed consent was obtained from both: participants and they parent and/or legal guardians, what is in agreement with law in our country.

### Statistical analysis

Categorical traits were described using integers and percentages. Numerical variables were depicted by their mean, median, standard deviation, 95% confidence interval, and minimum- to-maximum values. For contingency tables a chi-squared test of independency or Fisher’s exact test (for small cell numbers) were performed. The normality of distribution was tested using the Shapiro–Wilk W test. The homogeneity of variances was assessed by Levene’s test. A multifactor analysis of variance (for normally distributed variables) or generalized linear models (for non-normally distributed ones) were caried out to assess differences in numerical variables between the study groups. All the ANOVA and regression models were controlled for the study participants’ age and gender. Spearman’s correlation coefficient was computed in order to assess relationships between the extension and flexion angles. A level of p < 0.05 was deemed statistically significant. All the statistical procedures were performed by using IBM^®^ SPSS^®^ Statistics, version 28 (Armonk, New York, USA).

### Ethical approval

This study was performed in line with the principles of the Declaration of Helsinki 1964. The study has been approved by the Bioethical Committee of the institution of the last author (project number 8/2020).

### Consent to participate

Written informed consent has been obtained from a parent and/or legal guardian of participants included in the study. If patients were 13 years old or more the informed consent was obtained from both: participants and they parent and/or legal guardians, what is in agreement with law in our country.

## Results

Of the examined patients, 13 (11.6%) were classified as Type 1 based on the Watson Jones scale, 64 (57.1%) as Type 2, and four (3.5%) as Type 3. In addition, 31 (27.7%) were rated as Type 4: all accompanied by dislocation of the elbow joint. All patients with type I fractures were upper extremity athletes planning professional sport career.

In addition, 15 (13.4%) demonstrated minimal displacement of the fracture according to the Wilkins division, and 72 (64.2%) significant displacement. In four (3.6%) patients, the fragment was trapped in the elbow joint.

No significant difference in MEPS score was found between the dislocated and non-dislocated groups (Table 4). Of the 112 patients tested, 98 had an excellent treatment outcome, ten good and four moderate. Bone union was achieved in 111 patients. In the other patient, with an isolated fracture of the medial epicondyle, a symptomatic lack of bone union and fibrous union was observed; the medial epicondyle was displaced, but without pathological mobility. For this reason, a revision was performed, the fragments cleaned and stabilized with two pulling screws.

**Table 4:** Detailed Mayo elbow performance score divided by occurrence of joint dislocation (numerical traits) (n = 112).

Investigated trait	Type of fracture	Statistical parameter					*P-*value
		*M*	*Me*	*SD*	95% CI	Min–max	
Range of motion (point)	Dislocated	19.7	20	1.2	19.2–20.0	15–20	0.533
	Isolated	19.8	20	0.9	19.6–20.0	15–20	
Stability (point)	Dislocated	9.7	10	1.2	9.2–10.0	5–10	0.957
	Isolated	9.7	10	1.2	9.4–10.0	5–10	
Activities of daily living (point)	Dislocated	24.5	25	2.0	23.8–25.0	15–25	0.425
	Isolated	24.1	25	2.3	23.6–24.6	15–25	
Pain intensity (point)	Dislocated	43.1	45	5.1	41.2–44.9	30–45	0.793
	Isolated	43.3	45	4.7	42.3–44.4	30–45	
GLOBAL (point)	Dislocated	96.9	100	8.1	93.9–99.9	70–100	0.980
	Isolated	97.0	100	7.4	95.3- 98.6	65–100	

In the non-dislocated group (81 patients), a follow-up (mean 26.3 months) found the mean range of motion (ROM) for flexion in the elbow joint to be 140.7°, with an mean extension deficiency of 3.0°. Complete forearm pronation and supination (85°–90°) were noted in all 81 patients. In the dislocation group, the follow-up after 46 months found the mean flexion to be 134.5°, with mean extension deficiency of 6.1°. Both flexion and extension of the elbow were significantly more limited in dislocation group (p < 0.001 and p = 0.019, respectively) (Table 5); while one patient this group demonstrated a reduction in pronation and supination by 5°, the remaining patients presented a full range of pronation and supination.

**Table 5:** Characteristics of the study cohort by occurrence of joint dislocation (numerical variables) (n = 112).

Investigated trait	Type of fracture	Statistical parameter					*P*-value
		*M*	*Me*	*SD*	95% CI	Min–max	
Age (years)	Dislocated	11.9	12	2.6	10.9–12.8	5–16	0.669
	Isolated	11.6	12	3.4	10.8–12.3	3–17	
Observation period (month)	Dislocated	29.9	24	18.1	23.2–36.5	12–78	0.054
	Isolated	26.3	17	20.5	21.7–30.9	8–87	
Distance from the bone (mm)	Dislocated	25.2	25.5	9.2	19.9–30.5	8–39	** < 0.001**
	Isolated	16.9	8	45.4	4.5–29.3	0–250	
Extension (deg)	Dislocated	-6.1	-5	9.2	(-9.5)-(-2.8)	(-40)-5	**0.019**
	Isolated	-3.0	0	7.6	(-4.7)-(-1.3)	(-40)-5	
Flexion (deg)	Dislocated	134.5	140	11.9	130.2–138.9	100–145	** < 0.001**
	Isolated	140.7	140	5.8	139.4–142.0	120–145	
Valgus angle (deg)	Dislocated	8.8	8.5	4.0	7.3–10.3	0–15	0.255
	Isolated	9.7	10	3.7	8.9- 10.6	3–17	

Values marked bold are statistically significant.

The ulnar nerve function was normal in 110 patients, as assessed by the Froment test and sense of touch on fingertip V. The other two patients demonstrated finger V sensation disorders after the surgery; one required ulnar transposition due to long-term sensory disturbance and paraesthesia.

Valgus of the elbow ranged from 3° to 17° (mean 9.7°) in the non-dislocated group, and from 0° to 15° (mean 8.8°) in the dislocation group. This difference was not statistically significant (Table 5).

Among the group of fractures, seven cases demonstrated moderate instability of the elbow joint, indicating greater dynamic elbow valgus compared to the opposite limb. Five patients with an isolated fracture of the medial epicondyle and two patients with a fracture and accompanying dislocation of the elbow. Four of them reported the pain (Table 6).

**Table 6:** Valgus stress test—medial elbow instability.

Patients with an isolated fracture of the medial epicondyle. n = 5	Patients with a fracture and accompanying dislocation of the elbow. n-2	P value
2 unstable without pain	1 unstable without pain	0.6
3 unstable with pain	1 unstable with pain	

## Discussion

The optimal approach to treating fractures of the medial epicondyle remains a topic of debate; however, recent years have seen centres tighten the criteria for surgery^4, 13, 14^. Kammath et al. indicate that in the case of conservative treatment, the risk of complications due to a lack of bone union can increase by as much as nine-fold^4^. Pathy et al. claims that good outcomes have been achieved with nonoperative treatment for minimally displaced fractures^13^. Pezzutti et al. indicate in their systematic review that the most common cutoffs used by authors indicating the necessity for operative intervention were > 5 mm displacement^14^. Failure to achieve bone union is symptomatic in only 11% and is associated with painful elbow instability^11^; however, the remaining patients do not demonstrate symptoms^15^. In addition, Grahn et al. confirm that surgical treatment is the best option for displaced fractures accompanied by dislocation of the elbow^9^.

The most important factors influencing the decision to treat fractures of the medial epicondyle of the humerus are the degree of fracture displacement and ulnar nerve dysfunction. Hugans et al. report that neither sex, injury mechanism nor patient lifestyle play any role in treatment planning; however, age is significant^5^. In the present study, the decision to perform surgical treatment was based on the degree of fracture displacement according to the Watson–Jones scale: a precise classification system with a clear cut-off point. Patients with type 2, 3 and 4 fractures underwent surgery. However patients with a minimally displaced medial epicondyle fracture (Watson–Jones type 1) were operated for non-medical reasons in this study. Most often due to the pressure of the parents of children who train and practice upper extremity sports as professionals.

Another issue in this procedure is choice of implant material. Most of the surgeons choose one from two options. Cannulated screws or K-wires. According to Pezzutti et al. the majority of surgically treated patients were treated with cannulated screws (22%) or K-wires (59%)^14^. However Emir Q. Haxhija et al. claimed that they prefer fixation with two Kirschner wires for smaller fragments and in younger children. They use cannulated cancellous bone screws to stabilize apophyseal avulsions of the medial epicondyle in older children^16^. In our centres we preferred use K-wires on all patients regardless of age.

Baldini et al. compared two methods of fracture open reduction and internal fixation: with K-wires and magnesium screws. The results were similar in both groups in terms of: range of motion, alignment, pain, and MEPS, with excellent results in both groups^17^. Also Ergin et al. found in the comprise study that both method of fixation provide good results^18^.

The only disadvantage of the procedure is the degree of subjectivity associated with the assessment of the displacement on the X-ray images. Indeed, Pappas et al. show that even specialists in the field of Orthopedics and Traumatology assess the same radiographs differently^3^. Grahn et al. suggest that CT should be routinely performed for elbow assessment to better determine the degree of fragment displacement^9^. Based on our experience, X-ray images performed in two projections, AP and lateral, is sufficient for estimating the degree of displacement and deciding on further treatment. This also offers the added benefit of reducing the dose of ionizing radiation to which the child is exposed compared to the CT scan.

In our study, treatment outcomes were based on the Mayo elbow performance score. Out of 112 patients tested, 99 achieved an excellent result, nine good and four average. The findings reported by Tarallo et al., in which 100% achieved a perfect treatment result, seem unrealistic; in our experience, good or excellent results may be achieved by the vast majority of patients, but a 100% success rate is unlikely.

The high percentage of good treatment scores obtained in our study was not confirmed by Kammath et al., who report a lack of sufficient compression in fragments stabilised with two or three Kirschner wires and an additional brachio-palmar plaster dressing, applied for a period of four to six weeks depending on the age of the patient^4^. Our approach is confirmed by Shen et al. and Tarallo et al.^12, 19^ Limiting the range of motion in the elbow joint is the most important problem; as such, internal stabilization and activation is crucial^19^.

Our results for elbow ROM, as well as forearm pronation and supination, are satisfactory and do not differ from previous findings. Similar results were reported by Tarallo et al., where out of 33 operated patients, seven presented an extension limitation of 5 to 250, one a supination loss of 100, and two a flexion deficit of 50^12^.

Seven of the patients in the present study, viz*.* five with an isolated fracture and two with a fracture and dislocation, demonstrated moderate instability of the elbow and clinical features of UCL damage. All of the patient with elbow instability included in this study had significant displacement of the fragments (Watson-Jones III and IV). UCL injury with avulsion fracture of the medial epicondyle is rare, and is characteristic of younger children practicing sports such as baseball or tennis. The literature about postoperative valgus elbow instability is rather scare. Even in the high-number studies this problem is rare. For this reason statistical analysis showing differences between different methods of treatment or type of fracture and instability is difficult to perform^1, 2, 6, 7, 14, 17, 18^. In our series the instability was not related to the fact of elbow dislocation.

A more common problem in the aetiology of repeated injuries to the elbow is the isolated damage to the UCL, especially the anterior lobe^11, 20, 21^. As far as ulnar nerve disorders are concerned, they occurred in two patients (< 2%); one patient demonstrated sensory disturbances that resolved within a few weeks, while the other required nerve transposition.

Conclusions. ORIF with K wires is a safe procedure for treating medial epicondyle humeral fractures that yields good or very good results. Similar outcomes are observed between patients with and without dislocation according to MSPS; however, flexion and extension are more limited in the former group.

## Data Availability

The dataset generated during and analyzed during the current study are not publicly available. An anonymized version of the dataset is available from first author of the study (W.S.) on reasonable request.
